# Oxidation and electrical properties of chromium–iron alloys in a corrosive molten electrolyte environment

**DOI:** 10.1038/s41598-020-71903-0

**Published:** 2020-09-09

**Authors:** M. Esmaily, A. N. Mortazavi, N. Birbilis, A. Allanore

**Affiliations:** 1 Department of Materials Science and Engineering, Massachusetts Institute of Technology, Cambridge, USA; 2 School of Engineering and Applied Sciences, Harvard University, Cambridge, USA; 3 College of Engineering and Computer Science, The Australian National University, Canberra, Australia

**Keywords:** Materials science, Structural materials

## Abstract

Chromium–iron (CrFe) binary alloys have recently been proposed to serve as the “inert” anode for molten oxide electrolysis (MOE). Herein, the effects of anodic polarization on physical and functional properties of CrFe anodes in the corrosive environment of MOE are studied via empirical observations and theoretical calculations. The findings indicate that the alloys form an inner chromia–alumina solid-solution covered by an MgCr_2_O_4_ spinel layer. A survey into the electrical properties of the detected oxides suggests that the layered oxide scale function as an efficient conductor of electricity at elevated temperature. The formation mechanism of the oxides is also investigated.

## Introduction

Iron based alloys, including steels, remain the key structural materials for the numerous technologies that underpin modern civilization, be it pipelines, structures and transportation; however, the environmental implications of iron production are vast^1^. The major ironmaking technology employing blast furnaces is not only recognized as being energy intensive, but also emits large volumes of CO_2_^2–4^. This can be rationalized considering that it is the reducing power of carbon-containing species (e.g., CO to CO_2_ or C*n*H*m* to CO_2_ and H_2_O) that supports the reduction reaction of iron (Fe) oxide (e.g., Fe_2_O_3_ + 3CO → 2Fe + 3CO_2_)^5,6^. Through an integrated blast furnace production route, around 1.8 tonnes of CO_2_ are produced for every tonne of steel produced. This corresponds to > 2 billion tonnes of CO_2_ emissions annually, or 6–8% of the total anthropogenic greenhouse gas (GHG) produced worldwide^7^. Iron and steel production have come under amplified scrutiny over the last decades, however to date the efficiency of current ironmaking technologies is considered optimal, and recycling strategies have already been implemented^7,8^. Thus, there remains an imminent need to develop alternative metallurgical technologies that may substantially reduce or even eliminate CO_2_ emissions from iron and steel production.

Molten oxide electrolysis (MOE) for iron production is one candidate that is in the category of *ultra-low GHG emissions technologies* when driven by GHG-free electricity^12^. The chemical principle is the production of liquid iron from iron oxide, using the following electrolysis reaction of selective decomposition of Fe oxide (e.g., hematite here):

1 $${\text{Fe}}_{2} {\text{O}}_{3} (l) \, + \, 6{\text{e}}^{ - } \to \, 2{\text{Fe}}(l) \, + \, 3/2{\text{O}}_{2} (g)$$

**Figure 1:**
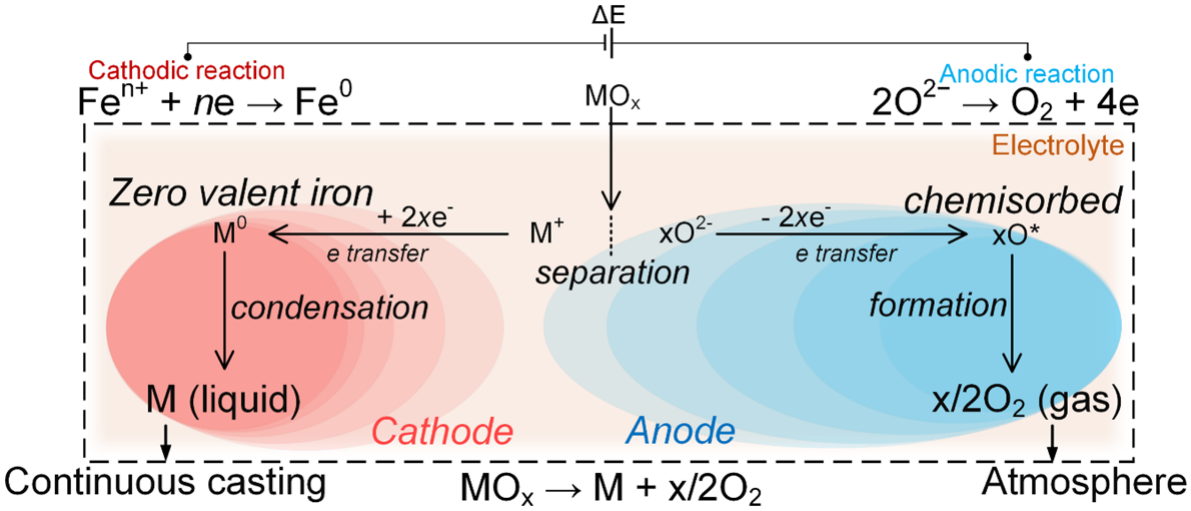
Illustration of the direct electrochemical conversion of a metal oxide (ore) to liquid metal and oxygen gas using electrolysis, which requires two-electrodes (a cathode and an anode) and an electrolyte.

The MOE process, if run above 1,535 °C can provide a liquid metal product, ready for steel production including continuous casting. Here, the sole by-product of the reduction reaction is oxygen gas, i.e., no CO, CO_2_, SO_2_ or NO_x_^6,9–12^. An historical and scientific perspective on the underlying progresses and challenges is available in the previous studies^13–15^. To date, MOE for ironmaking is a laboratory-proven extraction technology (Fig. 1)^12,15,16^, and is foreseen as scalable considering its analogies with molten salt electrolysis, which is used to produce several millions of tonnes of metals such as Al^17,18^, Mg^19,20^, Li^21,22^, Mn^23,24^ and rare earth metals^25–28^.

In traditional industrial electrolysis, an electrochemical cell is employed in which a direct current passes through a multi-element electrolyte, generating a cell voltage sufficient to drive an otherwise non-spontaneous chemical reaction (i.e., conversion of metal oxide to pure metal)^29–32^. A well-known example is the Hall–Héroult (H–H) cell, though to date this cell relies on a consumable carbon anode, leading to significant process CO_2_ emissions along other noxious species (CF_4_, C_2_F_6_, CO and SO_2_)^33–36^. Of course, significant power utilisation further adds to CO_2_ emissions in the case of smelters operating on electricity derived from fossil fuels, though such challenge is true across many materials production and does not pertain much to the issue of cell technology. As compared with the H–H cell and other molten salts routes, MOE involves generation of oxygen gas on an “inert” anode, in contrast to a consumable carbon anode^35–38^. Using a metal inert anode in electrolyte production—such as found for chlor-alkali—brings several process efficiency advantages, such as the ability to use a smaller anode–cathode distance or a lower ohmic drop in the anode section of the cell. Considering its underlying principles are long known, and the impressive list of potential advantages of MOE, its lack of demonstration outside of laboratories and at scale is surprising. Indeed, a number of critical materials related challenges require attention for success, as listed in Table 1^15,39–42^.

**Table 1:** Selected materials challenges related to the inert anode and electrolyte, in MOE^12,15,39–42^.

Cell component	Criteria to be met
Inert anode	Easily sourced and low cost
	Physically stable and mechanically robust at temperatures > 1,535 °C
	Resistant to corrosion by the multicomponent molten oxide (loss < 10 mm/year)
	Resistant to corrosion by oxygen gas (forming on the anode)
	Resistant to anodic polarization (current densities > 2 A/cm^2^)
	High electronic conductivity (to reduce ohmic-drop for current through anode)
	Resistant to thermal shock
Electrolyte (molten oxide)	Sufficient ionic conductivity (i.e., high diffusivity of reacting ions)
	Minimum corrosion towards the inert anode and the crucible
	Less dense than liquid Fe (density separation process of liquid iron)
	High solubility for Fe oxides (i.e., ability to act as solvent)
	Low vapor pressure at high temperature
	Absence of elements more noble than iron
	Melting temperature < 1,535 °C
	Environmentally safe and low cost

The electrolysis cell in MOE operates at temperatures above the melting point of Fe and involves the evolution of pure oxygen gas at atmospheric pressure at the anode. The design of each part of the cell, i.e., the container, the electrolyte, and the two electrodes, is subject to specific materials-related requirements (Table 1). Of these, an oxygen-evolving inert anode is the “Achilles heel” that restricts industrial implementation of MOE technology^12,42,43^. Candidate anode materials for large-scale development of MOE must fulfil several physical, chemical, and electrochemical characteristics. As detailed below, to date, only a few anode materials and designs have been proposed and few have been tested in laboratory experiments. Two main approaches have been proposed; one based on a solid, non-consumable, metal-based anode and another based on ceramics anode.

*The metal-based anodes* include platinum group metals (platinum, iridium (Ir)^15,44^, Ir-coated graphite^44^), and oxide-passivated Cr-based anodes^12^. While Ir-anodes have shown promising potentials, their large-scale use is limited by the high cost due to its extreme scarcity (~ 0.4 ppb in the Earth’s crust) and extreme hardness^13,40^. It is thus essential to develop lower cost anodes in order for MOE to become a viable alternative for steel production. Hence, rather than using Pt group elements, it would be preferable to utilise ‶inert‶ anodes made from base metal alloys and capable of forming thermally grown (TGO) protective oxide scales that meets the criteria listed in Table 1. This has been proposed and demonstrated in^12^ using a Cr-based metal anode, alloyed with Fe.

*The ceramic-based anodes* adopt a radically different approach. They include using magnetite (Fe_3_O_4_, the stable form of iron oxide at 1,535 °C in presence of > 7.2 × 10^–10^ atm of oxygen^45^) or ferrospinel-based ceramics such as Fe_3−x_Mg_x_O_4_^46^, Fe_3−x_Al_x_O_4_^47^, (Fe,Mg,Al)_3_O_4_^42,48^, (Fe,Mg,X)_3_O_4_ (X = Si and Zr)^43,49^. Those approaches rely on the limited solubility of the ceramics in the electrolyte, which is obviously a challenge in an electrolyte designed to dissolve sufficient amount of iron oxide for electrolysis. This approach has the elegance of potentially adding and using the feedstock iron oxide as a component of the anode, purposely consumed by the electrolysis process, potentially the lowest anode cost option possible. To maintain electrolysis conditions and in particular a reasonable cell voltage, the ceramics however need to exhibit high electrical conductivity, a key challenge for bulk ceramics above 1,300 °C in particular considering the need for high surface area of anodes for iron production, see e.g.,^50^. Those approaches push forward advanced ceramics able to perform mechanically, chemically and electrically in regions of high-temperature (ultimately close to 1,535 °C at the electrolyte), oxygen partial pressure (at some point pO_2_ needs to become close to 1 atm), gas evolution (more than 4 Nm^3^ of oxygen need to be removed per 1 tonne of iron) and high electric field. As evidenced by the recent literature, this is a frontier in engineered ceramics that calls upon the full tool-box of materials engineering.

To date, no evidence of successful oxygen evolution on ceramic-based anodes in the conditions of Fe production by MOE have been published, meaning the metal-based anodes are somehow more advanced. However, whilst acknowledging the efforts in the studies cited above, understanding the existing solution to the “ultimate materials challenge” offered by Cr-based anode remains to be completed. It is indeed required to push forward the materials understanding of the Cr-based anode oxidation and passivation performances during MOE. This is because the task of utilising metallic alloys as anode is not trivial since metal oxides are reported to be dissolved rapidly in molten oxides at temperatures > 1,050 °C^51,52^.

The present study fundamentally analyses the materials characteristics of the oxide layers found by Allanore et al.^12^, where Cr_1−*x*_Fe_*x*_ alloys were first proposed to be served as anode material in MOE. This alloy system spontaneously forms external layers of chromia (Cr_2_O_3_), which is a refractory ceramic and is utilised in a large number of applications including metal-oxide semiconductors, batteries, fuel cell electrodes, gas sensors, heterogeneous catalysts, and thermal barrier coatings (see^53–59^). Herein, we detail the corrosion and electronic properties of oxides forming on Cr_1−*x*_Fe_*x*_ alloys, when they are exposed to a molten calcium oxide-based electrolyte at 1,565 °C. The fundamental role of anodic polarization and electric current in the performance of the anode material was also investigated. In particular, an insight into the oxide layers formed during electrolysis is proposed, enabling some analogy and comparison with the ceramic-based anode findings. Experimental results obtained by several independent analytical tools, combined with thermodynamic calculations and considerations based on density functional theory (DFT) computations, are presented and discussed in detail.

## Methods

### Preparation of electrodes

**Figure 2:**
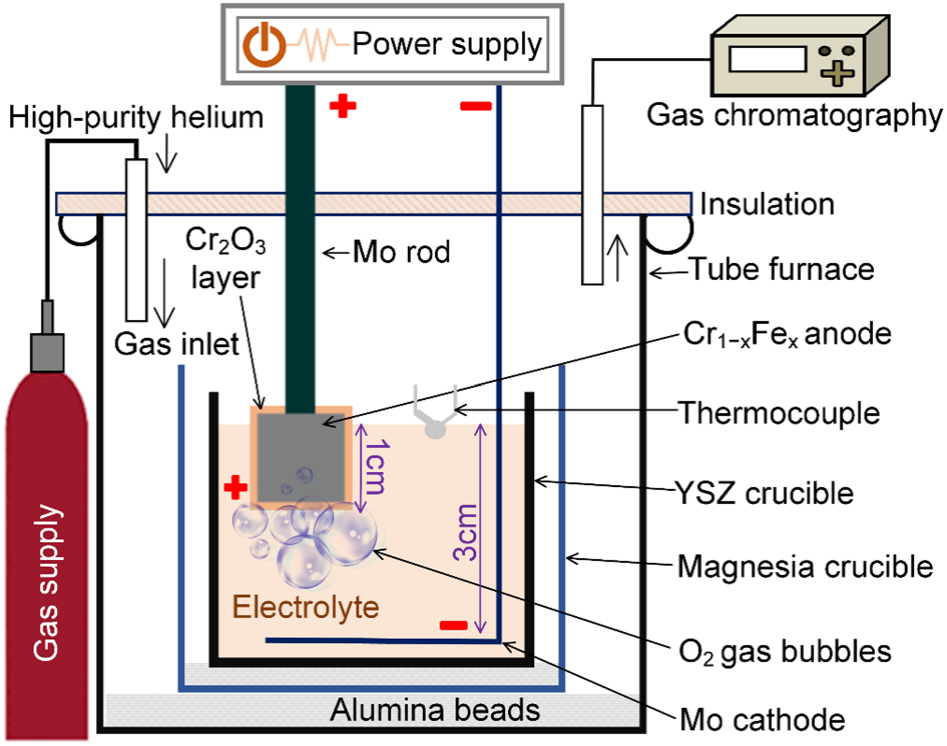
Schematic representation of the MOE apparatus illustrating the position of the anode.

The cathodes consisted of two Mo disks (10 mm thick and 38 mm in diameter). The anodes studied were binary chromium–iron alloys; Cr_1−*x*_Fe_*x*_ with *x* ranging from 0.1 to 0.3, supplied as strips produced using arc melting by Ames National Laboratory, Iowa, USA. The Cr–Fe alloys were machined to produce 2.0 × 1.0 × 0.7 cm^3^ anode specimens with a top pin of 1.0 × 0.5 × 0.7 cm^3^. The latter was then welded to a Mo rod electrode lead (see Fig. 2).

### Electrolysis and immersion experiments

Below are highlighted the essential materials and electrolysis details that supported the successful testing of the Cr-based anodes reported in^12^. Electrolysis and experiments were carried out in a vertical tube furnace (McDanel Advanced Ceramics) at 1,565–1,600 °C. The electrolysis cell was made of yttria-stabilized zirconia, itself contained in an outer magnesia (MgO) crucible (Tateho Ozark Technical Ceramics) as a precaution in case of failure of the inner crucible. The electrolyte selected for this study was composed of 42.3CaO, 42.3Al_2_O_3_, 5.4MgO and 10Fe_3_O_4_ (wt.%). Fe_3_O_4_ is added into the electrolyte as a surrogate iron oxide feedstock. Note that the physical and functional properties of molten oxide electrolytes as a function of their chemical composition are comprehensively investigated and discussed in^13,41^.

While the majority of the commercialized iron ore contain iron oxide as hematite (Fe_2_O_3_), Fe_3_O_4_ is the thermodynamically stable solid iron oxide phase at the target temperature (> 1,535 °C) and in an oxygen-rich environment (pO_2_ > 0.2 atm)^60^. The furnace tube was purged by a stream of high-purity He during the experiments, to preserve an inert atmosphere and enable detection of oxygen. The outlet gas was analyzed in situ using gas chromatography (CP-4900 Micro-Gas Chromatograph) (Fig. 2).

Galvanostatic was carried out at equivalent anode for a duration up to 6 h, which was restricted by the crucible material performance. The anode area used to evaluate the current densities is 1.4 cm^2^ and the current ranges from 2 to 9 A, delivered by a power supply Argantix XDS30-500-208IF. The experiments were performed in two steps: (i) pre-oxidization of the anode (at 1,450 °C for 2 h, in an equivalent partial pressure of oxygen estimated at pO_2_ = 10^–6^ atm) to form a protective Cr_2_O_3_ scale on the anode surface, and (ii) immersing the Mo cathode and subsequently the Cr_1−x_Fe_x_ anode under anodic polarization at a constant cell voltage of 2 V in the electrolyte melt, after which constant current electrolysis started.

### Post-experiment characterization

After the electrolysis experiments, the anode was sectioned and mounted in thermosetting polymer. To study the oxide layers formed on the anode, the samples were metallurgically prepared via grinding and polishing to a mirror-like surface finish, as described in^61–63^. The microstructures of samples were examined using an Olympus GX-71 optical microscope and a FEI Quanta 200 scanning electron microscopy (SEM) equipped with Oxford Link energy dispersive X-ray (EDX) microanalysis hardware. Quantitative metallography was performed to investigate the internal oxidation of the anode using the image analysis software ImageJ and Image Pro-Plus. A LEO Ultra 55 field emission gun SEM equipped with electron backscatter diffraction (EBSD) system was used to determine the crystal structure of the oxides^64,65^. Thin foil specimens for high-resolution microscopy were fabricated via the in-situ lift-out method in a FEI Versa 3D DualBeam focused ion beam/SEM (FIB/SEM) instruments equipped with OmniProbe micromanipulators. The scanning transmission electron microscopy (STEM) investigation was conducted using a FEI Tecnai T20 operating at an accelerating voltage 200 kV, equipped with a windowless Bruker EDX detector. STEM images were captured via the bright field (BF) and high-angle annular dark-field (HAADF) modes.

### Computational analyses

Calculations were performed to examine: (i) the thermodynamic stability of the various phases observed on the anode in the presence and absence of anodic oxygen gas, and (ii) the electrical properties of such phases. Thermodynamical calculations were performed using FactSage 7.2 software^66^. Calculations were performed at the electrolysis operating temperature (1,565 °C) and several databases were used including FactPS, FT oxid, FT salt, FT, CORU, and FT-AlSP. The Equilib module in FactSage was used to evaluate the stability of the identified oxidation products when Cr_2_O_3_ was in contact with the molten electrolyte. Some of the physical properties of the alloys were assessed using ThemoCalc software^67^.

First principles calculations were conducted based on the structural information of the detected phases and the chemical compositional experiments. In some cases, the software framework Materials Project, was utilised (Jain et al.^68,69^), in which density functional theory (DFT) calculations is performed as implemented in the Vienna Ab Initio Simulation Package (VASP) software. Calculations employed the generalized gradient approximation (GGA) and the GGA + *U* framework (*U* denotes an energy correction term to the *d* or *f* orbitals). The formalism GGA + U was introduced to solve the GGA-inherent limitations in considering the self-interaction errors and reaction energies faults when electrons are transferred between localized states (as in *d* or *f* orbitals in transition metal oxides). The GGA + *U* can be expressed as^70,71^:

2 $$E_{GGA + U} = jE_{GGA} + \frac{{\left( {U - J} \right)}}{2} \mathop \sum \limits_{\sigma } \left[ {\left( {\mathop \sum \limits_{j} \rho_{jj}^{\sigma } } \right) - \left( {\mathop \sum \limits_{j,l} \rho_{jl}^{\sigma } \rho_{lj}^{\sigma } } \right)} \right]$$

where $E_{GGA + U}$ is the total energy of the formulism GGA + *U*, $E_{GGA}$ is the total energy of GGA, $\rho$ is the occupation matrix of 3*d* orbitals with the subscripts $j$ denoting the *d*-orbital index and $\sigma$ indexing the spin. The parameters *U* and *J* (*U* in GGA + *U*) are the density matrix of *d* electrons and are the spherically averaged matrix elements of the screened Coulomb electron–electron interaction^68,69^. A drawback to the GGA + *U* approach is the correction energies cannot be directly compared with energies calculated via GGA due the correction addition of *U*. The software framework employed addresses this difficulty via breaking down reaction energies into component reactions: (i) best-represented in GGA, (ii) best-represented in GGA + U, or (iii) binary reactions that produce systems with localized states (e.g., oxides) from systems with delocalized electrons. The computations were performed at 0 K, 0 atm, in the absence of point defects. The accuracy of the calculations depends on the system examined. In case of reaction energies, where the reactants and products are all oxides, e.g., MgO + Al_2_O_3_ → MgAl_2_O_4_, calculation errors are smaller than that of reactions between chemically dissimilar systems (between metals and insulators)^68^.

## Results

### Compositional analysis of the reaction products after electrolysis

**Figure 3:**
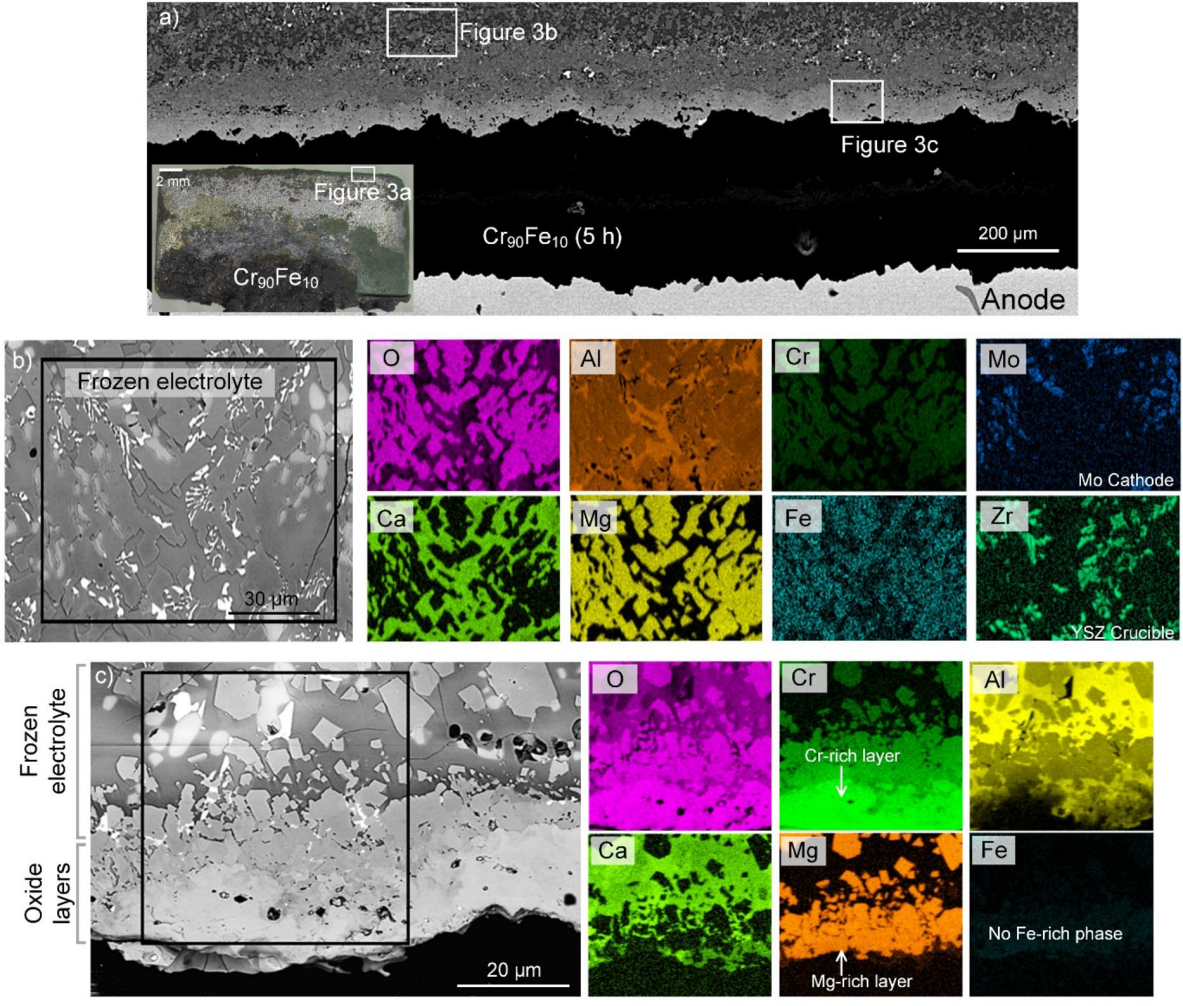
Overview of electrode cross sections. (**a**) A typical cross-sectional BSE-SEM image of Cr_90_Fe_10_ after 5 h electrolysis (the insert optical image is adopted from^12^ with permission from *Springer*), and (**b**,**c**) SEM–EDX analysis of two areas in the frozen electrolyte and close to anode surface after electrolysis. Note the presence of Ca- and Mg-rich phases in the frozen electrolyte and the Cr/Al oxide layer on the anode surface.

Three Cr_1−*x*_Fe_*x*_ anodes (*x* = 0.1, 0.2 and 0.3) were investigated after galvanostatic electrolysis experiments at 1,565 °C. Irrespective of electrolysis time and alloy composition, the alloys were completely covered by a layer of frozen electrolyte; see e.g., the overview image inserted in Fig. 3a. Cross-sectional micrographs and EDX analysis from the anode and the frozen electrolyte are shown in Fig. 3a–c. The frozen oxide electrolyte (with a thickness of ~ 1 mm) mainly was a Mg- and Ca-rich phases mixture. While the distribution of Al was relatively uniform across those phases, enrichment is observed in the Ca-rich phases (see the Al and Ca maps in Fig. 3b). The EDX analysis in Fig. 3b also indicates a small amount of Cr (< 0.5 at.%) in the Mg-rich phase. The Zr and Mo detected in the frozen electrolyte (Fig. 3b), present mainly in the Ca-rich phase, result from the corrosive action of the melt towards the zirconia crucible and the Mo cathode, respectively.

**Figure 4:**
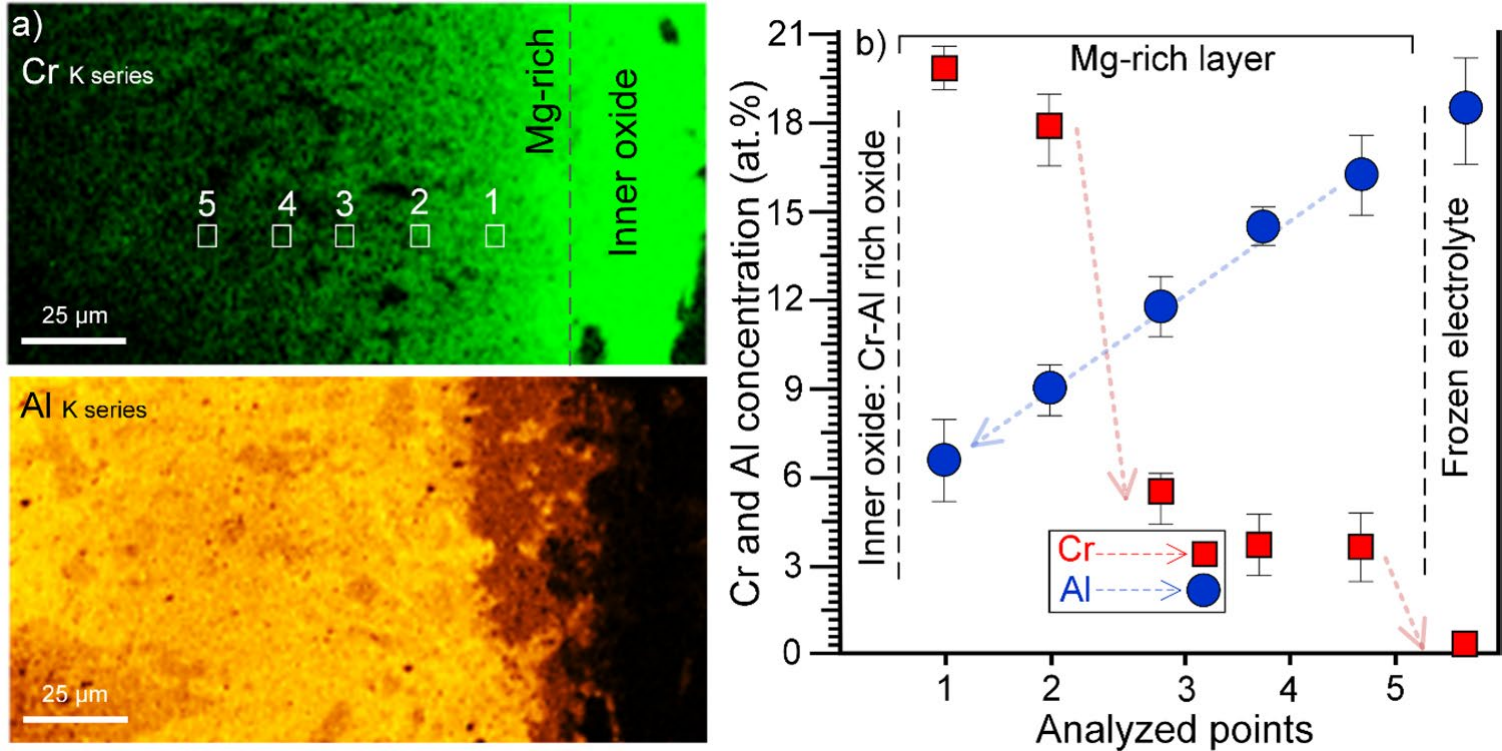
(**a**) The distribution of Cr and Al for the Mg-rich oxide layer formed upon the anode surface after 5 h electrolysis in the Ca-rich electrolyte at 1,565 °C, and (**b**) the plot showing the concentration of Cr and Al from the oxide layer towards the frozen electrolyte, based on quantitative EDX analysis.

Compositional analysis of an area on the anode surface (Fig. 3c) revealed the formation of a uniform oxide layer dominated by Cr and Al (see the O, Cr, and Al maps in Fig. 3c), likely corresponding to a solid-solution of Cr_2_O_3_ and Al_2_O_3_ (see below). This Cr/Al-rich oxide layer (hereafter the *inner* oxide layer) was separated from the frozen electrolyte by a layer containing Mg-rich oxides, which are further characterized in the following sections. Note that there was no evidence for Zr or Mo in either the Cr–Al oxide or the Mg-rich layer. Quantitative compositional analysis was conducted to study the distribution of elements in the frozen electrolyte, from the frozen bulk toward to its interface with the *inner* oxide layer. The Cr map and the corresponding EDX point analysis (see Fig. 4a,b) indicates that the Cr found in the frozen electrolyte is originated from the uniform inner Cr–Al-rich oxide layer on the anode surface. A steep gradient in Cr concentration is present from the anode’s surface towards the central parts of the frozen electrolyte. The electrolyte is lean in Cr (< 0.1 at.%) positions that were ~ 1 mm distant away from the anode surface. In contrast, the source for the Al in found in the oxide scale is the electrolyte, as presented in the Al map and the corresponding EDX point analysis in Fig. 4a,b.

**Figure 5:**
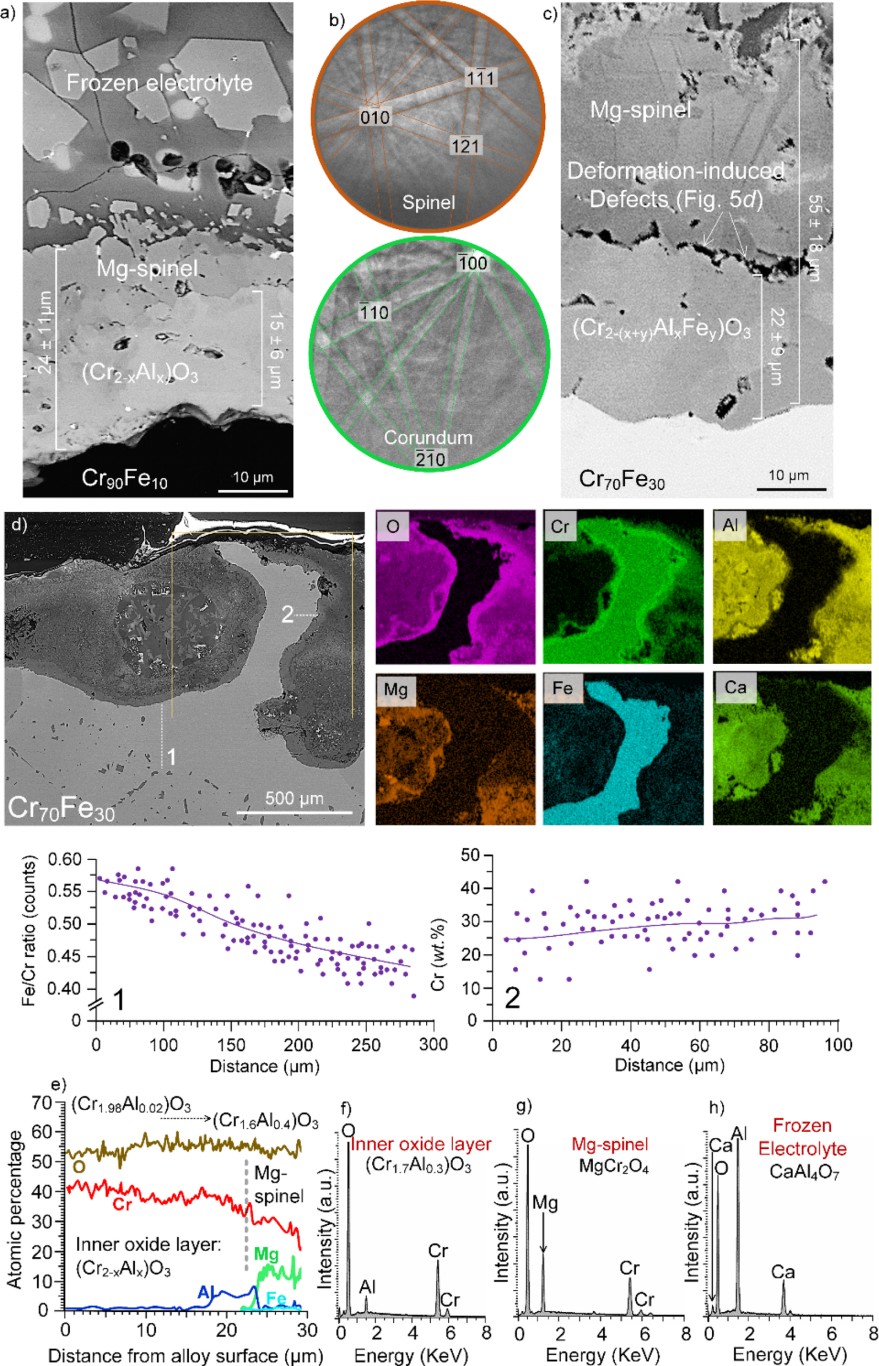
(**a**) Cross-sectional analysis (BSE-SEM image as well as EDX analysis) of the alloy with 10 wt.% Fe as well as the subsequent frozen oxide electrolyte, (**b**) EBSD Kikuchi patterns acquired from the *inner* Cr–Al *inner* oxide layer and the Mg-rich outer layer, revealing the corundum structure α-(Cr,Al)_2_O_3_ and the spinel structure of the Mg-rich layer, (**c**) cross-sectional image showing the oxide layers formed on the alloy with 30 wt.% Fe, (**d**) compositional analysis revealing an extensive Cr-depletion in the subsurface areas of the anode made of the Cr_70_Fe_30_ alloy, and (**e–h**) a quantitative EDX line scan from the inner oxide layer and EDX point analysis revealing the chemical compositional of the inner (Cr, Al)_2_O_3_ layer, the Mg-rich spinel layer on the anode surface as well as a Ca-rich phase in the frozen electrolyte.

Further compositional and structural investigations were performed on the Mg-rich layer and the *inner* oxide forming on the anodes with different chemical compositions using SEM-EDX and EBSD (Fig. 5). A typical cross-section of the oxides forming on the anode with the lowest Fe content (Cr_90_Fe_10_) as well as the frozen oxide electrolyte is shown in Fig. 5a. It is evident that the *inner* oxide layer (15 ± 6 µm) is thicker than the Mg-rich layer (12 ± 7 µm) for the Cr_90_Fe_10_ alloy. Phase identification of the *inner* oxide layer and the Mg-rich layer was performed using EBSD (Fig. 5b).

The Kikuchi patterns implied that the *inner* oxide adopts the corundum structure, with a trigonal symmetry (see e.g.,^72–74^), in accordance with the data reported by Allanore et al.^12^. According to the phase diagram^75,76^, at > 1,200 °C, the two oxides chromia (Cr_2_O_3_) and alumina (Al_2_O_3_) are completely miscible, forming stable solid-solutions.

It is key to note that the thermodynamically stable Al_2_O_3_–Cr_2_O_3_ solid-solutions are known for their superior mechanical properties and excellent thermal shock resistance, and hence, are widely utilized as corrosion resistant refractories in fibreglass furnaces, carbon black reactors, incinerators and wide range of solid waste vitrification processes as a corrosion resistance refractory^76–78^. The Mg-rich layer (Fig. 5b), formed uniformly on top of the *inner* layer at the oxide/electrolyte interface, adopts the spinel AB_2_O_4_ structure. Hereafter the Mg-rich layer will be referred to as “Mg-spinel”.

A typical cross-section of the oxides forming on the anode with the highest Fe content (30 wt.%) is shown in Fig. 5c. A comparison between the cross-sections in Fig. 5a,c implies that the thickness of the oxide/spinel surface layer formed on the Cr_90_Fe_10_ alloy is ~ 50% thinner than the alloy containing 30 wt.% Fe. Moreover, in the case of 30 wt.% Fe, the *inner* oxide layer with the corundum structure showed an insignificant amount of Fe (0.1–0.3 at.%) with the formula (Cr_2-(x + y)_Al_x_Fe_y_)O_3_. On the contrary, Fe was absent (or it was below the EDX detection limit), in the *inner* oxide layer formed on the surface of Cr_90_Fe_10_ during the electrolysis.

The three alloys (10, 20 and 30 wt.%Fe) also differed with respect to the composition of the Mg-spinel layer. The spinel layer formed on the 30 wt.%Fe alloy was lower in Al and higher in Fe and Cr than for the 10 wt.%Fe alloy. Also, a high density of voids was observed in the oxide layers formed upon Cr_70_Fe_30_, between the Mg-spinel and the inner oxide layer as well as within the (Cr_1−x_Al_x_)_2_O_3_ corundum-type oxide layer (Fig. 5c), which was not the case for Cr_90_Fe_10_ (Fig. 5a). The formation of voids cannot be attributed to cooling or to the volume change during the formation of the spinel from transient oxides, as discussed in^77^, because they were absent in the case Cr_90_Fe_10_. Instead the voids are suggested to form due to a rather significant deformation of the anode as a consequence of Cr-depletion in the alloy Cr_70_Fe_30_ (Fig. 5d). The thicker oxide scale (comprising the inner oxide and the Mg-spinel layer) and higher Cr content in the Mg-spinel layer on Cr_70_Fe_30_ correspond to a pronounced Cr-depletion of the alloy, compared to Cr_90_Fe_10_. Thus, the Cr content in the anode decreased to 40–50 wt.% after exposure (see the compositional variations the EDX line scan analysis in Fig. 5d).

Line scans and point analysis from the reaction products formed on Cr_90_Fe_10_ are depicted in Fig. 5e*–*h. Consistent with EBSD data, the analysed area closest to the anode surface is interpreted in terms of corundum-phase exhibiting solid solubility. The inner oxide layer thus consisted of a solid-solution of chromia–alumina; (Cr_1−x_Al_x_)_2_O_3_ (Fig. 5e). Similar to diffusion of Al in the spinel layer (Fig. 4b,c), the Al ingress into the Cr-oxide scale was proved to be gradual and diffusion-controlled (Fig. 5e). Thus, a range of compositions of the (Cr_2−x_Al_x_)O_3_ phase were detected in the inner oxide, from (Cr_1.98_Al_0.02_)O_3_ closest to the metal, to (Cr_1.85_Al_0.15_)O_3_ closest to the Mg-spinel layer and finally (Cr_1.6_Al_0.4_)O_3_ at the *inner* oxide/Mg-spinel interface (Fig. 5f). The corundum-type (Cr_2−x_Al_x_)O_3_ layer is in contact with a magnesium chromite (MgCr_2_O_4_) spinel (the point analysis "Mg-spinel" in Fig. 5g), following by the frozen electrolyte comprising Ca- and Mg-rich oxides (see the elemental maps in Fig. 3b,c as well as the point analysis in Fig. 5h). In some case, it was noted (not shown) that the Mg-spinel layer contains varying amounts of Al and Fe; (Mg_1−x_Fe_x_)(Al_1−x−y_Cr_x_Fe_y_)_2_O_4_.

**Figure 6:**
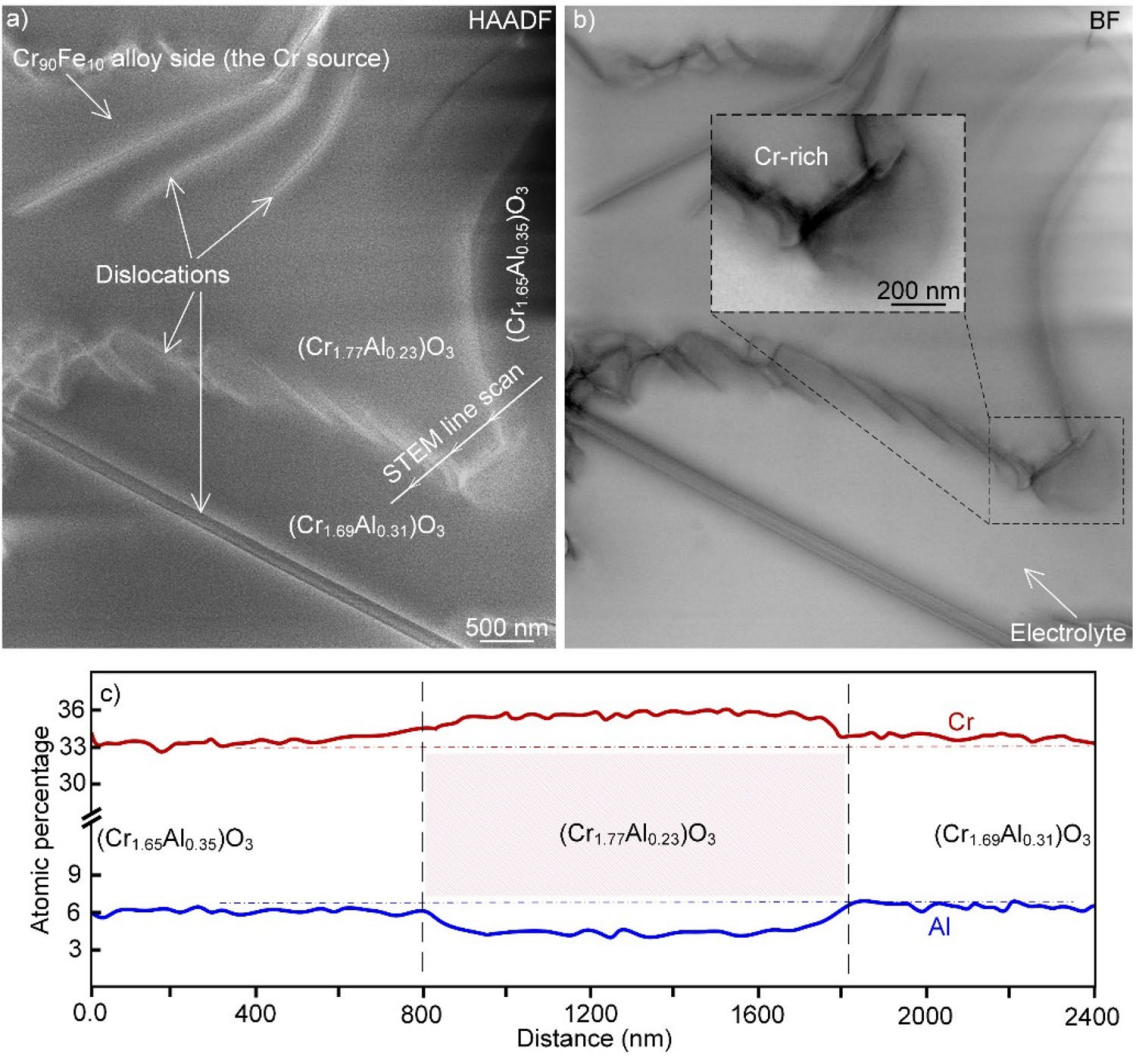
(**a**,**b**) HAADF- and BF-STEM images from an area in (Cr_2−x_Al_x_)O_3_, i.e., the inner oxide layer, revealing dislocation misfits at the interface of two regions with slightly different chemical compositions as well as the location of the alloy (Cr_90_Fe_10_) and electrolyte, and (**c**) a STEM line scan from the area shown in (**b**). Sample relates to 5 h electrolysis in molten electrolyte at 1,565 °C.

There is little information in the literature about the microstructure of the Cr/Al solid-solution oxides forming during exposure to a molten oxide electrolyte. Thus, to gain insight regarding the microstructure of the *inner* oxide layer, the corundum phase (Cr_2−x_Al_x_)O_3_, formed on alloy Cr_90_Fe_10_ during a 5-h electrolysis was further investigated using STEM imaging and STEM-EDX analysis on FIB-prepared thin foils (Fig. 6a–c). In the case of electrolysis, the *inner* oxide exhibited a uniform homogenous solid-solution microstructure with no evidence of precipitates e.g., no Mg-, Ca- and Al_2_O_3_–rich particles were found. This is an important result for the evaluation of the properties (e.g., electrical conductivity) of the *inner* oxide layer formed on the anode surface during electrolysis. Occasionally (Fig. 6a,b), "boundary"-type regions were observed, between two distinct regions with different chemical composition.

The boundary areas were noted to comprise an epilayer of misfit dislocations (as seen in the HAADF and BF micrographs in Fig. 6). STEM-EDX indicated that the region, which was closer the alloy substrate (anode) and separated by the misfit dislocations, contained more Cr with formula (Cr_1.65_Al_0.35_)O_3_ as compared with the surrounding matrix with an approximate formula (Cr_1.77_Al_0.23_)O_3_. It is noted that chemical composition of the region examined in Fig. 6 agrees well with what was detected using SEM-EDX (Fig. 5e). The results therefore imply that Cr-diffusion from the alloy substrate (anode) towards the molten electrolyte, appearing as the Cr-enrichment in the studied area, was accompanied by the formation of dislocations. It may be noted that it has recently been suggested that strain/stress in a solid-solution, sourced by the segregation-induced composition variations (as seen in Fig. 6c), drive the nucleation and migration of misfit dislocations^79^, somewhat similar to what is observed in Fig. 6a,b.

### Effects of current and alloy composition on the anode oxidation behaviour

**Figure 7:**
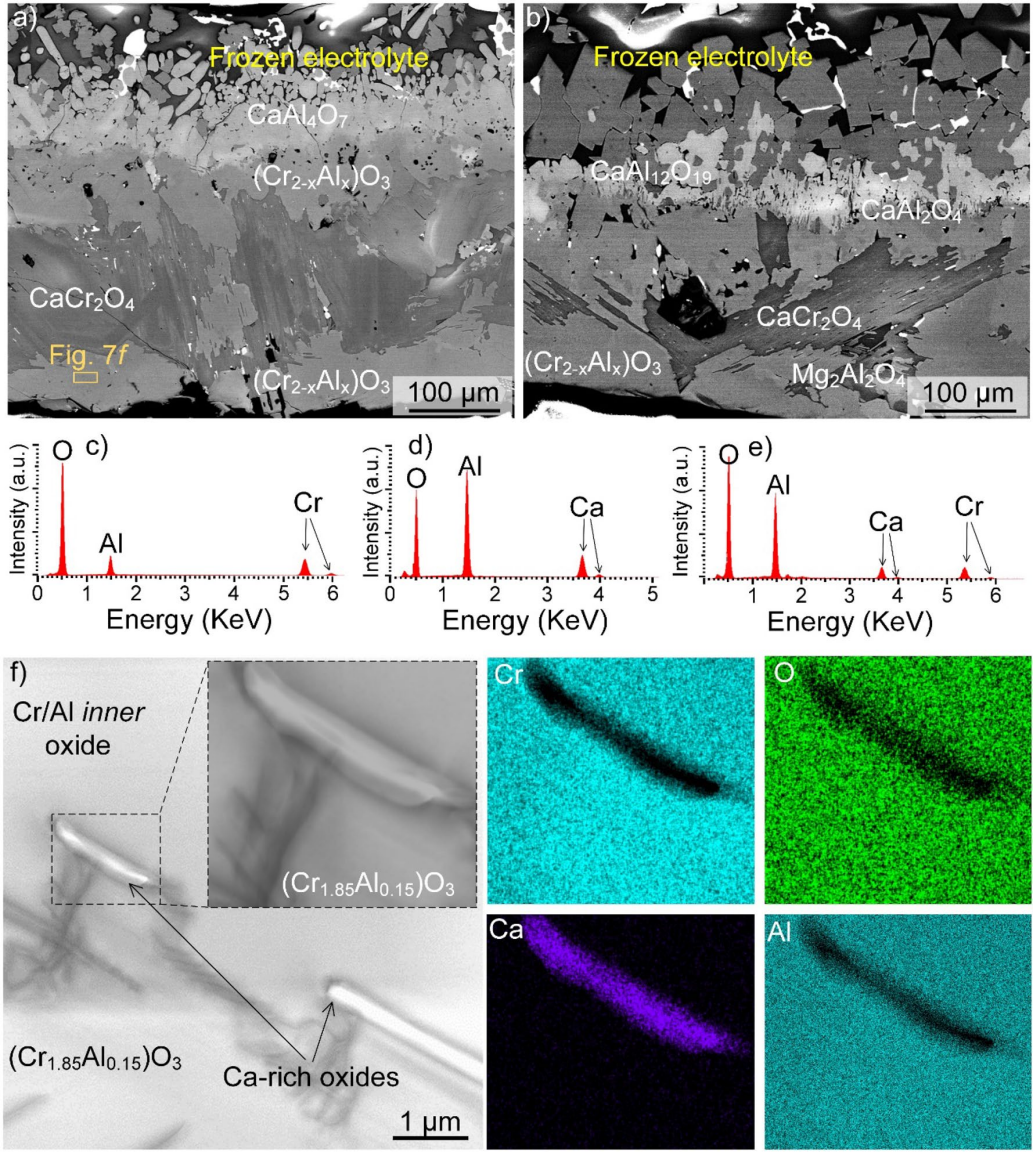
(**a**–**e**) SEM–EDX analysis revealing the oxide layers formed on Cr_90_Fe_10_ following immersion in the Ca-electrolyte for 5 h, and (**f**) BF-STEM micrograph and EDX elemental mapping from an area in the Cr/Al solid-solution formed on Cr_90_Fe_10_ during immersion experiment at 1,565 °C, revealing the presence of Ca-rich particles and thus the heterogenous nature of the inner oxide formed in the immersion condition. Note the formation of Ca-rich phases as opposed to the electrolysis case, where Mg spinels were the dominant phase.

The chemical composition of the oxide layers formed on the anode after static immersion in the molten electrolyte (i.e., in the absence of anodic polarization, electric current and oxygen evolution) at 1,565 °C was also investigated. Several cross-sectional micrographs and compositional data related to several area corresponding to various phases formed in this condition are shown in Fig. 7a–f. The results show that the morphology and chemistry of the reaction products are greatly different in absence of electrolysis conditions (compare Fig. 7a–e with Figs. 3, 4 and 5). Following electrolysis (Figs. 3, 4 and 5), the oxide scale exhibited a uniform *inner* layer consisting of a solid-solution of chromia and alumina, covered with a Mg-spinel MgCr_2_O_4_. In contrast, after immersion (Fig. 7), the corundum (Cr_2−x_Al_x_)O_3_ inner oxide layer was non-uniform, and the Mg-spinel MgCr_2_O_4_ layer was replaced by a layer dominated by Ca-rich spinels, especially CaCr_2_O_4_. STEM imaging and STEM-EDX (see e.g., Fig. 7f) also indicated that the inner Cr/Al-rich oxide layer contained sub-micron sized Ca-rich particles, indicating the microstructure of the Cr/Al rich oxide is not homogenous in the immersion condition, in contrast to that of the electrolysis case (Fig. 6a,b). Thus, anodic polarisation had a major effect on the microstructure and composition of the oxides forming on the anode surface.

Additionally, the scales formed during immersion (corundum and Ca-rich oxides) were thicker (160 ± 35 µm) than the scales (corundum and Mg-spinel) formed during electrolysis (90 ± 14 µm) in the case of Cr_90_Fe_10_. Moreover, the composition of the spinels depended on the distance from the anode surface. It was thus noted that Cr-containing spinels, MgCr_2_O_4_ in the case of electrolysis and CaCr_2_O_4_ in the case of immersion, were present close to the Cr-rich inner oxide, whereas Al-containing spinels (MgAl_2_O_4_ and CrAl_2_O_4_) were mainly formed close to the frozen electrolyte (see e.g., Fig. 7a).

**Figure 8:**
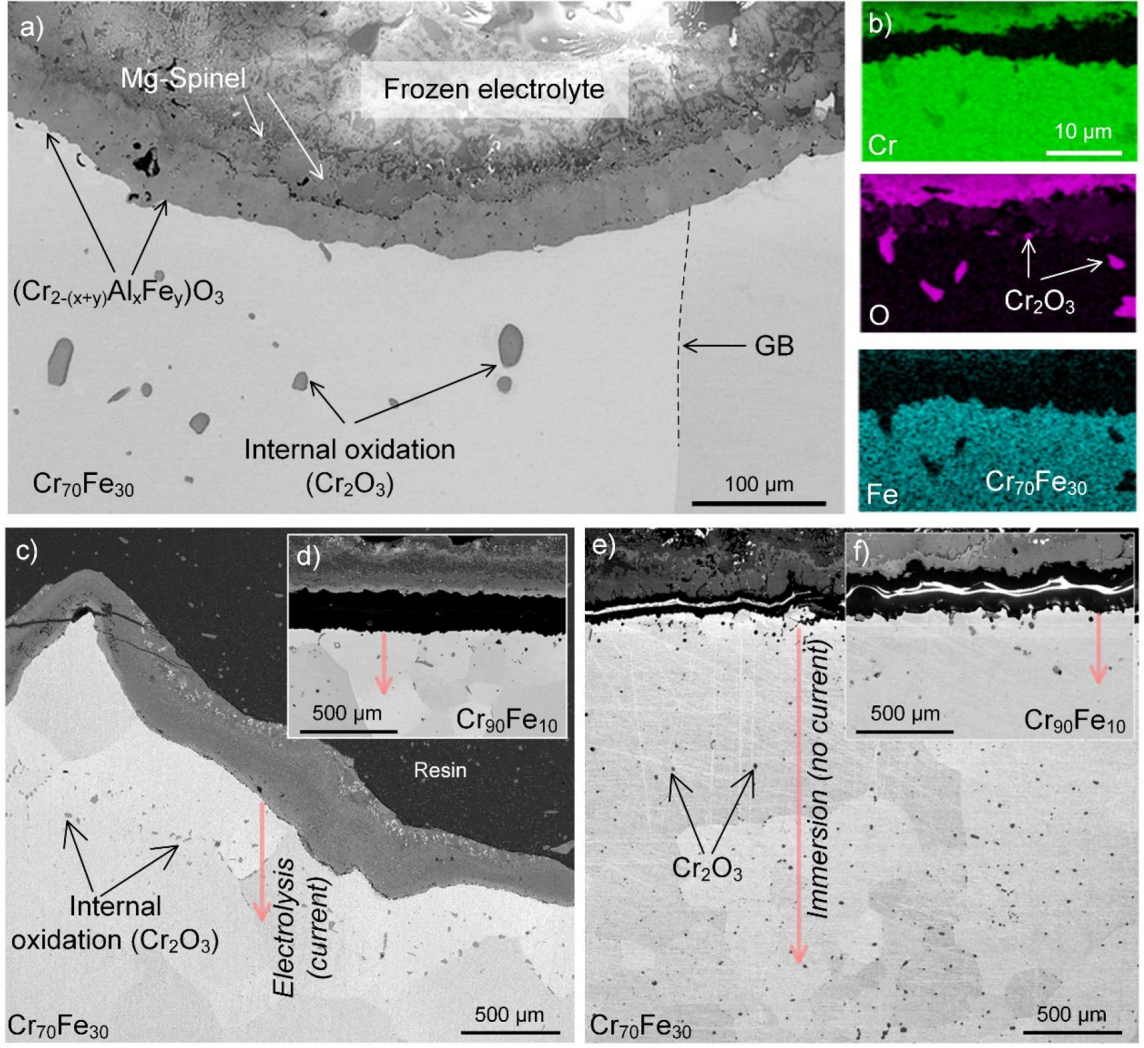
(**a**, **b**) SEM cross-sectional image as well as EDX data providing evidence for internal oxidation the formation of Cr_2_O_3_ in sub-surface regions of the alloy Cr_70_Fe_30_ after 5 h electrolysis at 1,565 °C, and (**c**–**f**) SEM-BSE images showing depths of internal oxidation in alloys with 10 and 30 wt.% Fe.

The effects of Fe content and electrolysis on the internal oxidation of the Cr-based anode were also studied, as shown in Fig. 8. In regards with the sub-surface areas (i.e., within the metal phase of the anode) influenced by the oxidation process, the term oxidation affected zone (OAZ), i.e., depth of the area exhibiting Cr-depletion and internal oxidation, is adopted. Internal oxidation appeared with the formation of Cr_2_O_3_ particles within the alloy (Fig. 8a,b). It was noted that OAZ was deeper in Cr_70_Fe_30_ than in Cr_90_Fe_10_ for both the electrolysis and immersion conditions (e.g., compare Fig. 8c,d). While the depth of internal oxidation in the Cr_1−x_Fe_x_ alloys was limited to 200–650 µm after electrolysis, an unlimited depth of internal oxidation was observed in Cr_70_Fe_30_ after 5 h of static immersion, i.e., in the absence of anodic polarization, as seen comparing Fig. 8c,d and Fig. 8e,f.

In contrast, the depth of the OAZ increased with electrolysis time in the case of Cr_70_Fe_30_ (not shown). Cr-depletion in the OAZ was much more pronounced under static immersion as compared with that of the electrolysis condition. The extensive depletion found in the immersion condition is attributed to the formation of Ca-based spinels itself and the faster diffusivity of Cr (+ III) in the Ca-based spinel than in the Cr/Al solid-solution^12^. Finally, it may be noted that the SEM images presented in Fig. 8 show that anode deformation was pronounced for the alloy with the highest Fe content, i.e., Cr_70_Fe_30_ (Fig. 8c), while the Cr_90_Fe_10_ was dimensionally stable (Fig. 8d).

### Thermodynamic considerations

**Figure 9:**
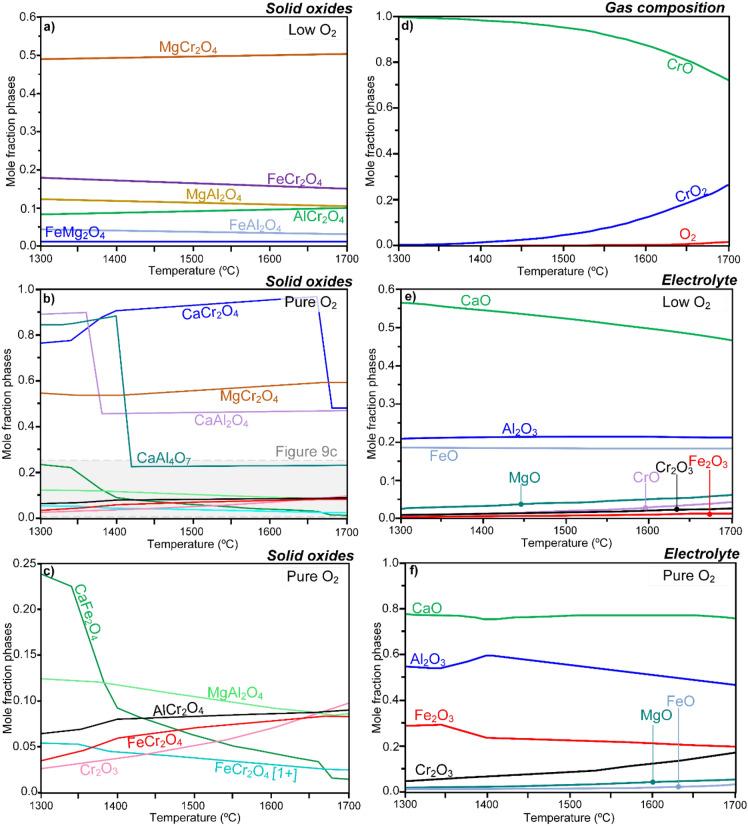
Thermodynamic calculations at different temperatures and different oxygen activities (i.e., oxygen content in the system) describing the relative stability of phases in the MOE condition. The phase diagrams correspond to the relative phase fractions of oxides: (**a**) at different temperatures and at low oxygen activity, and (**b**,**c**) different temperatures and at high oxygen activities, (**d**) gas composition, and (**e**,**f**) composition of the molten oxide electrolyte as a function of oxygen content and temperature. The PO_2_ varies over a relatively wide range in the electrolysis cell, from 1 atm at the anode surface to about 10^−9^ atm at the cathode.

Ample evidences are provided above indicating that the spinels forming during electrolysis are compositionally and characteristically different from those of the immersion experiments. In the next step, the formation of the detected oxides was rationalized using thermodynamic calculations and the results are presented in Fig. 9, where the relative phase fraction of each oxide for various oxygen gas partial pressure and temperature is described. Calculations were conducted at low and high partial pressures of oxygen at the temperature range relevant to electrolysis process. The possible formation of all the major solid oxides found post-experiment was accounted for in the calculations, which were carried out using the FactSage software coupled with the relevant thermodynamic databases (see the “Methods” section). Thermodynamics calculation were performed to predict the possible reactions when the molten electrolyte (CaO, MgO, Fe_3_O_4_ and Al_2_O_3_) is in equilibrium with Cr (+ III) oxide, which was formed on the anode surface due to the pre-exposure treatment of the Cr_1−x_Fe_x_ alloys prior to the electrolysis and immersion experiments.

The results (Fig. 9, where only the solid oxide phases are presented) suggest the formation and co-existence of the different Mg/Cr/Al and Ca/Cr/Al oxides in the studied environment, agreeing with the experimental findings such as the compositional analysis (Figs. 4 and 5). Indeed, all the main oxides detected experimentally (i.e., MgCr_2_O_4_, CaCr_2_O_4_, CaAl_2_O_4_ and CaAl_4_O_7_) to form on the anode surface during both electrolysis and immersion were also predicted by the calculations. At low partial pressure of oxygen (Fig. 9a), i.e., the case during static immersion, the equilibrium predictions showed that Mg-spinel MgCr_2_O_4_ is the dominant phase throughout the temperature range 1,300–1,700 °C. At high partial pressure of oxygen (Fig. 9b,c), resembling the condition during electrolysis, the relative mole fractions of the Ca-rich phases were dependent upon (i) temperature (Fig. 9b,c) and (ii) oxygen activity (content) (Fig. 9d). On the other hand, Mg-spinel MgCr_2_O_4_ proved to be barely dependent on (i) and (ii). Hence, as seen in Fig. 9b–d, while Ca-rich oxides (CaCr_2_O_4_, CaAl_4_O_7_ and CaAl_2_O_4_) were first the dominant phases at temperatures <  ~ 1,400 °C (Fig. 9b) and low oxygen activities of approximately < 10^–10^, the relative stability of Ca/Al-rich oxides started to drop for temperatures > 1,400 °C and oxygen activities of approximately > 10^–10^. Finally, based on the relative phase fraction of the oxides presented in Fig. 9b,c, only a very small amount magnetite (Fe_3_O_4_)-based spinels are present at high PO_2_, supporting the experimental data, where no Fe-containing spinels were detected on the anode surface after electrolysis.

Thermodynamic evaluations of the gas composition on top of the molten electrolyte as well as the Ca-rich electrolyte composition as a function of temperature at low and high partial pressures of oxygen are summarized in Fig. 9d–f. Thermodynamic predicts that only a trace amount of Cr_2_O_3_ is dissolved in the electrolyte, which is in accordance with the EDX data presented in Fig. 1b, and this occurs in the forms of Cr^2+^ and Cr^3+^, i.e., Cr + II and + III species, see Fig. 9d. The results thus suggest the absence of Cr in oxidation states > + III in the electrolyte. The phase diagrams presented in Fig. 9e,f also imply that Fe ions exist principally in the electrolyte as Fe^3+^ (Fe_3_O_4_ as the equivalent valence of Fe) close to the anode surface, where there is a high oxygen content, though as pO_2_ is reduced close to the cathode, more Fe^2+^ will become thermodynamically stable^14,50,60^.

### Electrical properties of the detected oxides

**Figure 10:**
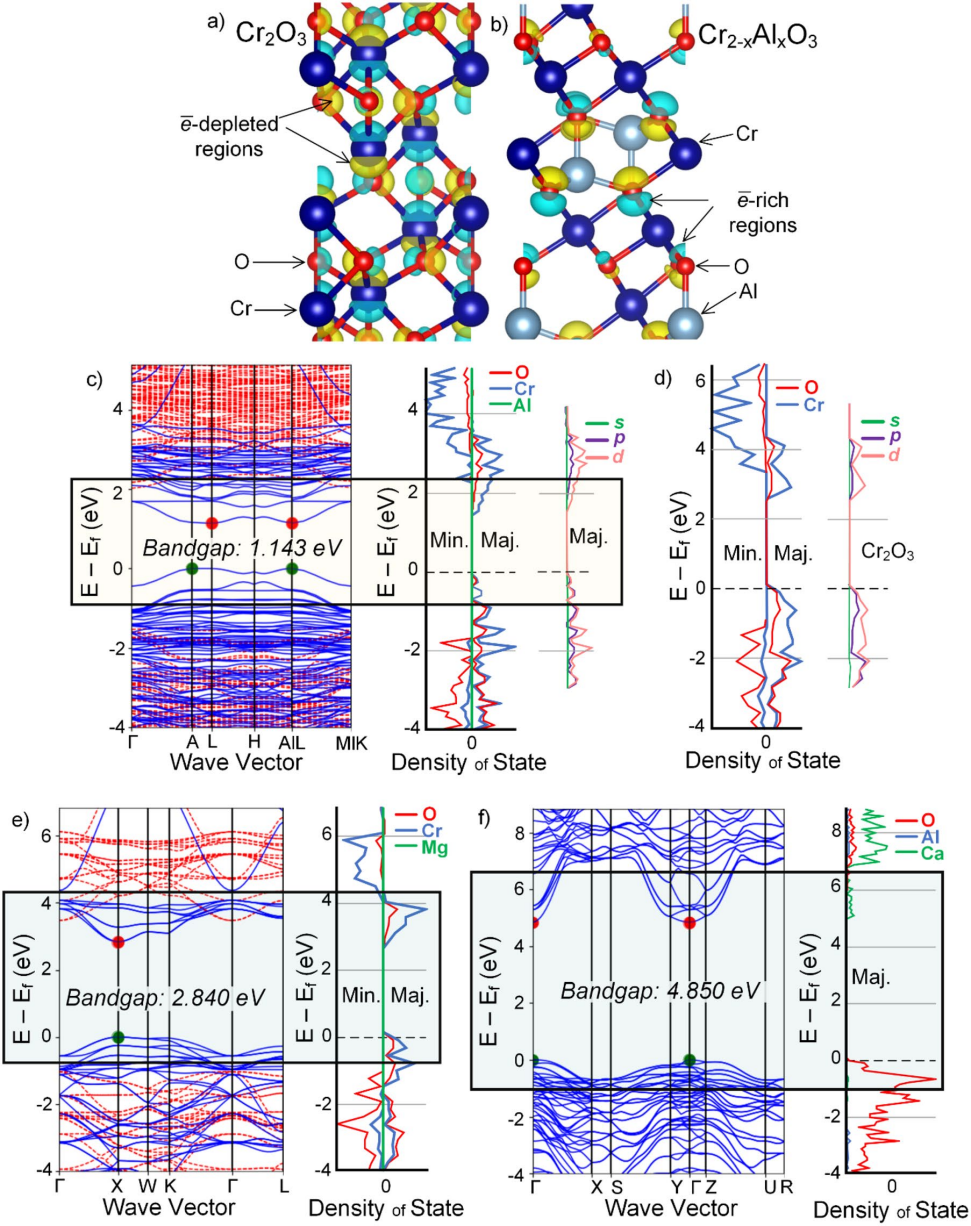
Investigating the electrical properties of the detected oxide. (**a**,**b**) The electronic structure of pure Cr_2_O_3_ and the experimentally detected chromia–alumina solid-solution, (**c**) the electronic band structure and DOS for chromia–alumina solid-solution, (**d**) DOS for pure Cr_2_O_3_, (**e**) the band structure and DOS for the Mg-rich spinel MgCr_2_O_4_ formed during electrolysis, and (**f**) the band structure and DOS for the Ca-rich spinel CaAl_2_O_4_ formed during immersion experiment. Note (1): The VB maximum in the band structures is set to be zero. Note (2): The band structure data is displayed with solid-blue lines for up spin and dashed-red lines for down spin. Note (3): The rectangles in the band structure and PDOS shows the area of interest, near the Fermi level. Note (4): In (**c**), The DOS data and band structure are slightly different as the *k*-point grid is not the same. Some of the results are generated by data from the Materials Project and pymatgen’s *electronic*_*structure* package, see^68,69^.

Understanding the electrical properties of the oxides formed during electrolysis is essential for an anode material to be functional in an electrolysis cell (Table 1). A too low electrical conductivity will increase the ohmic cost for the current flow, leading to an increase in the terminal cell voltage and a corresponding increase in energy consumption. Locally, a high ohmic drop can lead to local Joule heating that can also affect the temperature of the anode and its environment, ultimately affecting the thermo-mechanical stability of the anode or its protective. In this study, the crystal structure of the chromia–alumina solid-solution formed in situ during electrolysis was reproduced. A uniform and homogenous microstructure (as found in the STEM study, e.g., in Fig. 6), was adopted, and compared with that of α-Cr_2_O_3_. Note that the latter oxide is the anticipated oxidation product of the metal anode in absence of molten oxide and electrolysis. The structures were calculated using a spin-polarised plane wave DFT calculation as implemented in VASP. As indicated above, GGA + U method with Perdew Burke Ernzerhof (PBE) functional were used. Calculations presented in Fig. 10a,b verified the persistence of the original (Cr_2_O_3_) corundum crystal structure, consistent with EBSD (Fig. 5b).

In the structures, blue color represents electron-rich and yellow color represents electron-depleted regions. In the Cr_2_O_3_ lattice, the O atoms follow an *hcp* stacking, while the Cr atoms exhibit an *abcabc* stacking sequence as found in face center cubic (fcc) lattices^80,81^. The atomic position of Cr in the solid-solution is altered when compared to Cr_2_O_3_ due to the addition of Al atoms into the system. Thus, the clusters of electrons in the starting Cr_2_O_3_ structure are shared uniformly throughout the lattice, and become highly concentrated in the regions near the Al atoms. In Cr_2_O_3_, the Cr–O bond length is 2.03 Å, while in the solid-solution, the Cr–O bond length ranges from 1.93–2.09 Å and the Al–O bond length is 1.83 Å. In Cr_2_O_3_, O is shared with 4 Cr atoms (1 configuration), while in the solid-solution, O is shared with 3 Cr/1 Al atom and with 3 Cr atoms (2 configurations). The charge separation between electron-rich and electron-depleted regions are changed from initially centralised in Cr atoms in Cr_2_O_3_ into O atoms in the solid-solution. The negative charge in the Cr/Al oxide is concentrated more towards Al and the positive charge is concentrated more towards Cr. The electronic configuration in α-Cr_2_O_3_ (Fig. 10a) is thus significantly altered in the presence of Al.

The computed band structure and density of states (DOS) for some of the main reaction compounds with the compositions detected experimentally are also presented in Fig. 10b–e. As opposed to the chromia–alumina solid-solution, the electrical properties of Cr_2_O_3_ (with a calculated formation energy of -2.31 eV) is well studied in the literature^80–84^. It is a wide bandgap oxide and reported bandgap energy range from 2.8 to 3.2 eV^80,82^. In the band structures shown in Fig. 10, the difference between the maximum of the valence band (VB) and the minimum of the conduction band (CB) defines the bandgap energy, and the energy levels next to the Fermi level are of interest, shown as rectangles in Fig. 10. The DOS profiles project on the orbital show the total energy of electrons for the elements in the detected oxides. In the case of chromia, the DOS is also provided (Fig. 10c). The results showed that the detected chromia–alumina solid-solution (with a formation energy computed to be ~ − 2.55 eV) has a smaller bandgap energy than pure chromia, indicating that the solid-solution is electrically more conductive than pure Cr_2_O_3_. Based on the peaks related to the total energy of Cr and O, it is suggested that the Al atoms in the chromia lattice lowers the migration energy of Cr atoms through altering the characteristics of *p*-, and in particular, *d*-orbitals.

The spinels detected experimentally exhibit larger bandgap energy (2.84 eV for MgCr_2_O_4_ and 4.85 for CaAl_2_O_4_) (Fig. 10d,e) than the inner oxide layer composed of Cr/Al solid-solution (1.14 eV). Comparing the Mg-rich and Ca-rich spinels, the former is found to exhibit a smaller bandgap energy. It may also be noted that chromia and the chromia–alumina solid-solution (Figs. 10b) shows indirect bandgap, i.e., the momentum of electrons and holes is the same in both the CB and VB, whereas the Mg- and Ca-spinel (Fig. 10c,d) exhibit indirect band gap, i.e., the *k*-vectors are different. While this is important for the light emitting characteristics of the oxides, it is difficult to make a direct link between this feature and the electrolysis conditions at the moment.

There are also differences between the band structure and DOS of the Cr- (Fig. 10a–d) and the Al-containing oxides (Fig. 10d) due to the unique electronic properties of Cr_2_O_3_ and Al_2_O_3_. For example, In the Al–Ca spinel, the VB electronic states are dominated by O (*p* orbitals) with insignificant contributions from Al (*p* and *s* states). In contrast the dominant contribution to the VB electronic states is from Cr in Fig. 10b,c. Besides, the bottom of the CB shows a greater curvature, as opposed to the Cr-rich oxides, with a nearly parabolic shape at the Γ point, somewhat similar to the band structure of α-Al_2_O_3_^85,86^.

## Discussion

### Anode oxidation behavior

In MOE environment, the formation of the (Cr_1−x_Al_x_)_2_O_3_ solid-solution is remarkable not only because for its ability to form a protective oxide scale on Cr_1−x_Fe_x_ alloys but also for the oxide solution electrical properties. At 1,600 °C, the calculated diffusion rates of Cr^3+^ in such solid-solution, using the compositions detected experimentally, are in the range 4.5–9.2 × 10^–10^ cm^2^/s; much lower than in pure chromia (1.05 × 10^–8^ cm^2^/s), demonstrating its enhanced corrosion resistance as compared with pure chromia. After the pre-oxidation treatment (see experimental), the Cr_1−x_Fe_x_ anode was covered by an adherent uniform chromia layer. The chromia–alumina solid-solution can form by reaction of the chromia layer with the aluminate-containing molten electrolyte. Ion exchange is among the reaction paths that may be involved:

3 $${\text{Cr}}_{2} {\text{O}}_{3} ({\text{s}}) \, + {\text{xAlO}}_{2}^{ - } ({\text{melt}}) \, \to {\text{Cr}}_{2 - x} {\text{Al}}_{x} {\text{O}}_{3} ({\text{s}}) + {\text{xCrO}}_{2}^{ - } ({\text{melt}})\quad 0.02 < x < 0.4$$

Contact adsorption of aluminate ions on the electrode surface is essential for the reaction (Eq. 3) to proceed. Thus, while ion exchange according to Eq. (3) is expected both under immersion conditions and electrolysis, ion exchange could be enhanced in the latter case assuming the aluminate ions are attracted by the anodically (positive) polarized electrode surface.

The findings presented herein suggest that the oxidation of the Cr_1−x_Fe_x_ anode is kinetically controlled not only by the solid-solution Cr_2−*x*_Al_*x*_O_3_(s) oxide but also by the Mg-spinel MgCr_2_O_4_, which forms as a continuous layer in the corrosion front (Figs. 3 and 5). Indeed, an appreciable decrease in rate of corrosion of Cr-based refractories has also been reported in the literature because of the formation of the Mg-rich spinel phases, see e.g.,^87–89^. This scenario agrees well with the observation that the depth of the oxidation affected zone in the alloys was much smaller during electrolysis (Mg–Cr spinel) than during immersion tests (Ca-rich oxides), compare Fig. 8c with Fig. 8e.

Thermodynamic (Fig. 9) predicts that the Ca-rich oxides become unstable, i.e., do not form, at high temperatures and high PO_2_. In contrast, in high partial pressure of oxygen, e.g., in the electrolysis case, MgCr_2_O_4_ is predominant, in accordance with the data presented herein. It is hence appropriate to discuss all the possible formation pathways of this important reaction product, detailed in Table 2.

**Table 2:** Possible corrosion reactions resulting in the formation the MgCr_2_O_4_ on the anode surface.

Equation	Description	No.
Cr_2_O_3_(s) + *x*MgAl_2_O_4_(s) → *x*MgCr_2_O_4_(s) + (Cr_1−x_Al_x_)_2_O_3_	Based on doping of the Cr_2_O_3_ layer	Eq. (4)
MgAl_2_O_4_(s) + 2CrO_2_^−^ (melt) → MgCr_2_O_4_(s) + 2AlO_2_^−^ (melt)	Reaction of MAS with CrO_2_^-^ species	Eq. (5)
Cr_2_O_3_(s) + MgO(melt) → MgCr_2_O_4_(s)	Reaction of Cr_2_O_3_ with MgO	Eq. (6)
CaCr_2_O_4_(s) + MgAl_2_O_4_(s) → MgCr_2_O_4_(s) + “CaAl_2_O_4_(s)”	“Metathesis reactions”/chemical exchange	Eq. (7)

In Eq. (10), the reaction product CaAl_2_O_4_ is placed inside “double primes” as the stoichiometry might be different.

One scenario is that MgCr_2_O_4_ forms due to the Al doping of the Cr_2_O_3_ (Eq. 4). However, since the amount of aluminium in the solid-solution oxide scale is small to match the amount that is found in magnesium chromite, this is not likely the major reaction. It is possible that the Mg–Al rich phases, e.g., magnesium aluminate spinel (MAS), MgAl_2_O_4_, which form close to the molten electrolyte play a major role, as described in Eq. (5). In this case, the relative activity of CrO_2_^−^ and AlO_2_^−^ in the melt becomes important. It is, however, noted that the formation of the Mg-spinel via Eq. (5) is highly unlikely as Cr species of oxidation states > III are not stable in the electrolysis condition, see the thermodynamic calculations in Fig. 9d–f. Additionally, a parallel reaction can be the reaction of the Cr_2_O_3_(*s*) scale with MgO(*l*) from the melt, as in Eq. (6). This reaction would depend on the activity of MgO in the melt, in particularly next to the anode. Finally, MgCr_2_O_4_ could also form as a result of the so-called *metathesis* reaction (Eq. 7), which has close to zero change in entropy and therefore has to have a negative reaction enthalpy to be spontaneous.

### Electrical properties of the oxide scales

DFT considerations (Fig. 10) showed that the Cr_2−x_Al_x_O_3_ has a smaller bandgap energy than α-Cr_2_O_3_ due to the altering role of Al on the geometrical as well as electronic structure of Cr_2_O_3_ (Fig. 10a,b). This is consistent with the results reported by Chapman et al.^90,91^, who experimentally measured the conductivity of co-precipitated chromium–alumina catalysts. They noted that the additions of alumina for up to 10–15 at.% gives rise to an appreciable increase in the conductivity of Cr_2_O_3_. This is due to the significant alterations in the electronic structure of the oxides and also due to the mismatch induced into the chromia lattice as well as the generation of ionic defects through the shrinkage of the octahedral sites by smaller Al^3+^ ions (octahedral ionic radius, *r*_*oct*_ 553.5 pm) replacing Cr^3+^ (*r*_*oct*_ 561.5 pm) in the chromia–alumina solid-solution^92^. It has previously been hypothesized, based on careful experimental measurements, that the lattice in chromia–alumina solid-solution contains a high fraction of defects, e.g., large number of stacking faults (see the work performed by Pedersen et al.^93^). Yet, it is important noting that our computation results on to the electrical conductivity of the oxides (Fig. 10) are 0 K calculations, neglecting the temperature effect. The relationship between temperature and the electrical conductivity can be expressed by^94^:

8 $$E_{G} (T) = E_{G} (0) - \frac{{\alpha T^{2} }}{(T + \beta )}$$

where $E_{G} (0)$ is the bandgap energy; the limiting value of the bandgap at 0 K, *T* is temperature, and $\alpha$/$\beta$ are constant values to fit the experimental data. As seen in Eq. (8), the bandgap energy decreases as temperature increases due to expansion in the crystal lattice expands. In addition, the increased temperature leads to availability of more charge carriers as well as weakening of the interatomic bonds, which in turn results reduces the required energy to break the bond, and transferring an electron in the conduction band. Based on experimental works, at temperature > 1,000 °C, in a wide range of oxygen partial pressure, chromia exhibits an electronic (*p*-type) conductor behavior, meaning that the valence and conduction bands overlap^80,81^. This allows electrons to flow through the lattice with a negligible energy penalty. A similar temperature-dependence is very well anticipated to exist for Cr_2−x_Al_x_O_3_ (0.02 < *x* < 0.0.4), i.e., the main oxide forming during electrolysis.

### Effect of current (or anodic polarization)

The results discussed above show that anodic polarization does not result in the dissolution of the oxide scale in the molten electrolyte due to the stability of the identified oxides. However, anodic evolution of oxygen with electrolysis is found to be a key factor in the resultant morphology and composition of the oxide layers on the metal surface. This realization is based on the experimental observation (e.g., Figs. 3 and 5) on the formation of two distinct layers (Cr_2−x_Al_x_O_3_ and the Mg-Cr-rich spinel) during electrolysis, and, the formation of Ca-rich spinels on the anode surface during immersion. This phenomenon, i.e., the current/polarization-induced changes in the characteristics of the oxide layer, could be considered in an analogy to the known effects of electric current on the formation of barrier oxide layers on aluminium alloys during anodization^95^.

It has been suggested that current alters the charge transfer of ions at the solid oxide scale/molten electrolyte interface, whereby the increased current-induced incorporation of electrolyte anions combined with the electro-migration of cations (Ca^2+^) can be considered as the reason for the absence of Ca-rich oxides^12^. In this study, a new explanation is provided based on thermodynamic calculations (Fig. 10). Hence, it was noted that Ca-rich mixed oxides are not thermodynamically stable, thus do not form, as the oxygen activity increases on the anode surface, where oxygen bubbles are generated via the half-cell O_2_ evolution reaction:

9 $${\text{O}}^{2 - } ({\text{electrolyte}}) \, = \, \raise.5ex\hbox{$\scriptstyle 1$}\kern-.1em/ \kern-.15em\lower.25ex\hbox{$\scriptstyle 2$} {\text{O}}_{2} ({\text{g}}) \, + \, 2{\text{e}}^{ - } ({\text{anode}})$$

Instead, thermodynamics predicts that Mg-rich spinels are the stable compounds in conditions with high partial pressure of oxygen, which is consistent with the experimental observations. This indicates that, at high oxygen activity, calcium chromite (+ III) reacts with oxygen and is electrochemically oxidized at the anode surface (Eq. 10):

10 $${\text{CaCr}}_{2} {\text{O}}_{4} ({\text{s}}) \, + \, 4{\text{O}}^{2 - } ({\text{melt}}) \, \to {\text{ Ca}}^{2 + } ({\text{melt}}) + \, 2{\text{CrO}}_{4}^{2 - } ({\text{melt}}) \, + \, 6{\text{e}}^{ - }$$

### Effect of alloying

The Fe content of the alloy influenced the composition, morphology, thickness and in general the performance of the oxide layers both during electrolysis and under no current conditions. The alloy Cr_90_Fe_10_ proved to be best performing, followed by Cr_80_Fe_20_ and Cr_70_Fe_30_. The reduced oxidation resistance of the alloys with increasing Fe content is attributed to the significant depletion of Cr in the sub-surface region of the anode, which cause for example a lowering of the melting point. Based on two commercial databases in ThermoCalc software, an alloy with composition Cr_40_Fe_60_ (Fig. 5d) melts in the temperature range 1,552–1,566 °C. This explains the observed reshaping (by diffusion) and deformation of Cr_70_Fe_30_ in e.g., Figs. 5d and 8c, which was not observed in the case of Cr_90_Fe_10_. Anode deformation causes the uniform oxide layer to fail as well as formation of interfacial defects and extensive internal oxidation in the alloys with high Fe content (as described in Fig. 8).

### The need to develop new alloys for MOE

While the above discussion shows that the double layered oxide scale formed on Cr_90_Fe_10_ fulfils many of the requirement for anode in MOE (Table 1), there remains a need to further enhance the efficiency of the MOE system through improving both the oxidation and electrical properties of the alloy. It is in particular interesting to investigate the oxidation of Cr–Fe-based alloys as well as the electrical properties of the oxides forming in this environment when the candidate materials are alloyed with trace amounts of (< 0.1 at.%) of the so-called reactive elements (REs) such as Ce, La, Y, and Zr. From an oxidation standpoint, it is well-established that the protective character of the oxide layer formed upon high-temperature alloys can be improved by several orders of magnitude by the small addition of RE elements though changing the oxide growth mechanism and improving the scale adherence to the alloy surface^73,87^. The electrical conductivity of the solid-solution oxide is also expected to be optimized further through increasing the number of impurity centres (RE-doping) that are known to facilitate the formation oxygen vacancies in the lattice e.g., via lowering the formation energy for Cr vacancies in the chromia lattice^95,96^. However, the effect of RE elements (REE) on the characteristics of chromia and alumina scales have only been studied at temperatures below 1,100 and 1,300 °C, respectively^57,73,87,97^. This means that the role of REs on the characteristics of the oxides formed upon anode in the MOE’s operating condition at temperatures exceeding the melting point of Fe, and in general on the oxidation performance of the Cr alloys and the electrical properties of the respective oxides, should also be explored in the futures studies.

## Conclusions

In summary, an improved understanding of the oxidation characteristics and electrical properties of Cr_1−x_Fe_x_-based anodes and the oxides formed during the electrolytic production of iron/steel by molten oxide electrolysis was achieved in the study presented herein. The main conclusions of the present study are as follows:

- During anodic polarization, the alloys developed a protective surface scale of chromia–alumina solid-solution Cr_2−x_Al_x_O_3_ (0.02 < *x* < 0.0.4). This "inner" oxide scale was covered by a "corrosion front" layer composed of Mg-spinel oxides, MgCr_2_O_4_ being the principal reaction product. The formation mechanism of the two oxides were discussed in detail, considering all possible reaction in the environment and operating temperature of MOE.
- Increasing the Fe content in the alloy was noted to result in substitution of Cr_2-x_Al_x_O_3_ inner oxide layer by (Cr_2-(x + y)_Al_x_Fe_y_)O_3_. The alloy containing 30 wt.% Fe (Cr_70_Fe_30_), was observed to develop a thicker and more defective oxide scale than Cr_90_Fe_10_. This resulted in a substantial Cr-depletion, altering the alloy’s physical properties.
- In the absence of any applied electric current, Ca-rich oxides dominated. The more protective character of Mg-rich spinels, formed during electrolysis, was evident experimentally as compared with that of Ca-rich oxides. Assessing the thermodynamic stabilities of the oxides as function of oxygen partial pressure (PO_2_) demonstrated that PO_2_, induced by the electric current during electrolysis, play a decisive role in the resultant chemical composition of the spinel phases.
- Thermodynamic calculations indicated that MgCr_2_O_4_ is the stable oxide at high partial pressure of oxygen and at high temperatures, i.e., the condition close to the anode surface, which agreed well with the empirical observations. Thermodynamic also confirmed that Cr with oxidation states > III are not stable at the MOE’s temperature and that the iron ions species have a marginal role in the overall stability of the solid oxide layers.
- A survey of the electrical properties of the experimentally detected oxides/spinels using DFT calculations provided information regarding the electronic structure of the oxide layers formed on the surface of the anode. The chromia–alumina solid-solution was determined to possess desirable electrical conductivity as compared to other detected oxides. Additionally, the Al-rich spinels (e.g., CaAl_2_O_4_), which dominate the surface oxide layer in the absence of electric current, display electrical properties close to that of Al_2_O_3_ (i.e., the insulating oxide).

The findings presented herein expand the knowledge of the performance of passivating alloys anodes and help the current understanding reaching a level of maturity such that it is possible to plan designing new metallic-based anodes. It is stressed that many electrolytic-based technologies could be benefited greatly by developing inert anodes using more accessible materials, i.e., oxide passivating alloys.

## Data Availability

The raw/processed data required to reproduce these findings cannot be shared at this time as the data also forms part of an ongoing study.
